# Multiple Functions of KBP in Neural Development Underlie Brain Anomalies in Goldberg-Shprintzen Syndrome

**DOI:** 10.3389/fnmol.2019.00265

**Published:** 2019-11-01

**Authors:** Hsin-Yun Chang, Haw-Yuan Cheng, Ai-Ni Tsao, Chen Liu, Jin-Wu Tsai

**Affiliations:** ^1^Institute of Brain Science, School of Medicine, National Yang-Ming University, Taipei, Taiwan; ^2^Brain Research Center, National Yang-Ming University, Taipei, Taiwan; ^3^Department of Biological Science and Technology, National Chiao Tung University, Hsinchu, Taiwan

**Keywords:** kinesin-binding protein (KBP), KIF1BP, KIAA1279, Goldberg-Shprintzen syndrome, cortical development, neuronal migration, dendritic aborization, axonal growth

## Abstract

Kinesin-binding protein (KBP; KIF1BP; KIAA1279) functions as a regulator for a subset of kinesins, many of which play important roles in neural development. Previous studies have shown that KBP is expressed in nearly all tissue with cytoplasmic localization. Autosomal recessive mutations in KIAA1279 cause a rare neurological disorder, Goldberg-Shprintzen syndrome (GOSHS), characterized by microcephaly, polymicrogyria, intellectual disability, axonal neuropathy, thin corpus callosum and peripheral neuropathy. Most *KIAA1279* mutations found in GOSHS patients are homozygous nonsense mutations that result in KBP loss-of-function. However, it is not fully understood how KBP dysfunction causes these defects. Here, we used *in utero* electroporation (IUE) to express KBP short hairpin RNA (shRNA) with green fluorescent protein (GFP) in neural progenitor cells of embryonic day (E) 14 mice, and collected brain slices at different developmental stages. By immunostaining of neuronal lineage markers, we found that KBP knockdown does not affect the neural differentiation process. However, at 4 days post IUE, many cells were located in the intermediate zone (IZ). Moreover, at postnatal day (P) 6, about one third of the cells, which have become mature neurons, remained ectopically in the white matter (WM), while cells that have reached Layer II/III of the cortex showed impaired dendritic outgrowth and axonal projection. We also found that KBP knockdown induces apoptosis during the postnatal period. Our findings indicate that loss of KBP function leads to defects in neuronal migration, morphogenesis, maturation, and survival, which may be responsible for brain phenotypes observed in GOSHS.

## Introduction

Goldberg-Shprintzen syndrome (OMIM 609460) (GOSHS) is an autosomal recessive congenital anomaly syndrome characterized by multiple dysfunctions in both the central nervous system (CNS) and the peripheral nervous system (PNS) (Goldberg and Shprintzen, 1981). Classical characteristics of GOSHS include intellectual disability, microcephaly, dysmorphic facial characteristics, and Hirschsprung’s disease (Hirst et al., 2017). *KIAA1279*, located on human chromosome 10q21.1, has been identified as the causative gene of GOSHS (Brooks et al., 2005). So far, seven *KIAA1279* mutations have been reported in GOSHS patients, most of them being homozygous nonsense mutations (Salehpour et al., 2017). Different mutations on *KIAA1279* may also show distinct phenotypes, including polymicrogyria and thin corpus callosum, suggesting diverse functions of KIAA1279 in neural development (Drevillon et al., 2013; Salehpour et al., 2017).

*KIAA1279* encodes for the kinesin-binding protein (KBP; also known as KIF1-binding protein, KIF1BP), which was originally found to interact with KIF1C and other kinesin-3 family members (Wozniak et al., 2005). KBP was also shown to interact with SCG10, a neuron-specific microtubule destabilizing protein in mouse neuroblastoma cells and zebrafish (Alves et al., 2010). In human fibroblasts, KBP interacts with the microtubule and actin cytoskeleton, and may function as an actin microtubule cross-link protein (Drevillon et al., 2013). Recently, pull-down assays combined with mass spectrometry showed that KBP binds to the motor domain of specific KIFs, such as KIF1A-C, 3A, 13B, 14, 15, and 18A, and modulates neuronal cargo transport as well as microtubule dynamics (Kevenaar et al., 2016). These studies provided insights into the molecular functions of KBP in regulating kinesins and the cytoskeleton.

Previous studies using cell lines, zebrafish, and mouse models have shed some light on the functions of KBP in neurons. KBP knockdown decreased neurite length in PC12 cells and SH-SY5Y (human neuroblastoma) cells, while overexpression of KBP increased neurite length (Alves et al., 2010; Drevillon et al., 2013; Kevenaar et al., 2016). Overexpression of KBP also reduced axon length in cultured mouse hippocampal neurons, and KBP mutants disrupted axonal microtubules, defecting axon outgrowth and maintenance in zebrafish (Lyons et al., 2008). In addition, KBP, as a kinesin regulator, is important for assuring correct functions of kinesins, which play a fundamental role in neural development by controlling cargo transport and microtubule organization. Therefore, dysregulation of kinesins due to KBP dysfunction may result in abnormal neural development and activities (Kevenaar et al., 2016). Recently, CRISPR-Cas9 mediated KBP-knockout mice have been created to investigate the effects of KBP loss-of-function on the nervous system. Mice lacking KBP underwent perinatal death and exhibited smaller brains, olfactory bulbs and anterior commissures, as well as defects in vagal and sympathetic innervation of the gut (Hirst et al., 2017). However, the cellular mechanism of KBP dysfunction that causes these defects remains unclear.

In the current study, we aimed to investigate the roles of KBP in brain development and its cellular mechanisms responsible for brain anomalies in GOSHS. During early cortical development, the self-renewing progenitors, i.e., radial glial cells (RGCs), produce neurons through asymmetric division (Hansen et al., 2010; Jiang and Nardelli, 2016). The newborn neurons then migrate radially to the cortical plate (CP) and differentiate into pyramidal projection neurons, which comprise 80–90% of cortical neurons. At this stage, these cells also generate an axon that extends into the lower intermediate zone (IZ), the area which becomes the future white matter (WM) (Hatanaka et al., 2004; Arikkath, 2012; Cooper, 2014). Using *in utero* electroporation (IUE) of short hairpin RNA (shRNA) to knockdown KBP in neural precursors, we directly investigate KBP functions in the mouse cerebral cortex and demonstrate that KBP is required for neuronal migration, maturation, morphogenesis, and cell survival. Moreover, our results bring us to look deeper into the cellular mechanisms of KBP-dependent cortical dysfunctions in GOSHS.

## Materials and Methods

### Constructs

The targeting sequences of KBP shRNAs were chosen from a portion of the KBP-coding region using BioSettia shRNA-Designer and was validated to avoid off-target knockdown by NCBI Nucleotide BLAST tool (shKBP-1: GGAAATAGAGGTTGAGTTA; shKBP-2: GAGATACTTGA GGCCCTTAGA; shKBP-3: GCTCAAGTCTACCAGCACATG; shCtrl: CCGGTCCTAAGGTTAAGTCGCCCTCGCTCGAG CGAGGGCGACTTAACCTTAGGTTTTTG). These sequences were inserted into pLKO-TRC011 [with green fluorescent protein (GFP)] vectors (National RNAi Core Facility at Academia Sinica, Taiwan). Plasmids used for IUE were prepared using the MaxiPrep EndoFree Plasmid kit (Qiagen). For KBP expression in the cell lines, Mouse 2510003E04RIK ORF mammalian expression plasmid, C-GFPSpark tag (Sino Biological Inc.; Catalog Number: MG51968-ACG; RefSeq: NM_02817.2) was used. For expressing KBP in the animal model, KBP-cDNA (NM_02817.2) was digested from Mouse 2510003E04RIK ORF mammalian expression plasmid and inserted into the PCIG2-IRES-GFP vector by using Xhol1 and EcoR1 restriction enzymes. HA was tagged at the N-terminus. PCIG2-IRES-GFP construct containing CAG promoter is a generous gift from Dr. Olivier Ayrault, Institute of Curie (Chang et al., 2019; Chen et al., 2019). Mutagenesis experiment (QuikChange Lightning Site-Directed Mutagenesis Kit, Agilent Technologies) was performed to change four sites of nucleic acids on the shKBP targeting region of the KBP sequence, introducing silent mutations to create shRNA-resistant mouse KBP.

### Primary Cortical Neuron Culture and Lentiviral Transduction

Cortical neurons were prepared from E14.5 mouse embryos via papain dissociation system kit (Worthington, Catalog Number: LK003153) as described previously (Huettner and Baughman, 1986; Lu et al., 2018). Neurons were cultured in Neural Basal Medium/L-glutamine/B27/penicillin/streptomycin (Cold Spring Harbor Protocols). Lentivirus carrying KBP shRNAs or scramble sequence was transduced into primary cortical neurons after 3 days *in vivo* (DIV 3) and harvested at DIV7 and DIV9. All shRNA reagents and lentivirus particles were purchased from the National RNAi Core Facility at Academia Sinica, Taiwan.

### Electrophoresis and Western Blotting

The cell lysates were collected by lysing cells under RIPA buffer (50 mM Tris–HCl, pH 8.0, 150 mM NaCl, 1% NP-40, 0.5% sodium deoxycholate, 0.1% SDS) and mixed with protease inhibitor cocktail and phosphatase inhibitor cocktail (Roche). Collected proteins were quantified by BCA protein assay (Pierce). Next, electrophoresis was performed on 30% acrylamide/bis-acrylamide gradient gel (TOOLS biotechnology), and then transferred to a polyvinylidene difluoride (PVDF) membrane (Millipore) with a transfer apparatus (Bio-Rad). PVDF membranes were then incubated for an hour in 5% milk and 1% BSA in 0.1%TTBS buffer (0.1% TWEEN20 in TBS buffer) for blocking, and then incubated in primary antibodies overnight at 4°C. Primary antibodies including anti-KBP antibody [Proteintech, Catalog Number: 25653-1-AP, 1:500; Santa Cruz, KBP Antibody (H-12): sc-390440], anti-GFP antibody (Abcam, 1:5000), anti-HA antibody (Proteintech, 1:500), anti-Pro-Casepase3 antibody (1:1000), anti-Cleaved-Casepase3 antibody (1:500), anti-MAP2 antibody (GeneTex, 1:100), anti-tubulin alpha antibody (Proteintech, 1:5000), and anti-beta-actin antibody (Proteintech, 1:5000). The immunoblots were then washed in 0.1% TTBS buffer for 10 min three times, and then immersed in the second antibody solution including goat anti-mouse IgG-HRP and goat anti-rabbit IgG-HRP (Santa Cruz) for 2 h. The immunoblotted proteins were visualized by an enhanced chemiluminescence ECL reagent (Santa Cruz) and detected by Luminescence Imaging system LAS-4000 (Fujifilm). The intensity of the band was analyzed by ImageJ (NIH).

### *In utero* Electroporation

*In utero* electroporation was performed as previously described (Chen et al., 2018, 2019; Lu et al., 2018). Briefly, all surgeries were done on pregnant ICR (BioLASCO) mice at E14.5, and were anesthetized by Isoflurane. The abdominal area was shaved and cleaned with alcohol. We then made a 3 cm incision through the skin and abdominal muscle to expose the underlying viscera. The uterine horns were carefully externalized. 1 μl solution containing DNA plasmid (1–5 μg/μl) with Fast green (2.5 mg/ml, Sigma) was injected into the lateral ventricle of the brain and electroporated using electrode forceps (5 mm in diameter; Harvard Apparatus). The forceps provided five electric pulses separated by 450 ms intervals at a voltage of 40 V. The uterine horns were put back into the abdominal cavity and the incision was closed. The embryos were harvested 4 days after electroporation or at postnatal day 6 and 12 for further experiments. Embryos and infant mice were perfused with PBS and fixed by 4% paraformaldehyde (PFA). Brains were collected immediately and immersed in 4% PFA for 12 hours for post-fixation, and then suspended in PBS solution.

### Immunofluorescence Staining

For brain slice staining, fixed brains were embedded in 4% low-melting-agarose (Amresco, United States) dissolved in PBS and sectioned coronally on a Vibratome (Leica), with 100 μm thickness for each slice, and stored in PBS with 0.05% sodium azide (Sigma) at 4°C. Brain slices were incubated in a blocking buffer (10% normal goat serum, 5% BSA and 0.2% Triton X-100 in PBS) and shaken at room temperature for one hour, followed by primary antibody immersion at 4°C for over two nights. Slices were washed in PBS for 10 min three times and then incubated with the secondary antibodies at room temperature for 2 h.

For cultured neurons staining, cells were fixed in 4% PFA in PBS and incubated at 37°C for 15 min, and then placed in 0.1% TritonX-100 diluted in PBS for permeabilization. After incubation in blocking buffer (5% normal goat serum, 5% BSA and 0.1% Triton X-100 in PBS) for 30 min at room temperature, cells were placed in primary antibodies for 2 h at 4°C, washed in PBS, and incubated with the secondary antibody for 1 h at room temperature. The primary antibodies used for immunostaining were: rabbit anti-Pax6 (BioLegend, 1:500), rabbit anti-ki67 (Millipore, 1:600), rabbit anti-NeuN (Millipore, 1:500), rabbit anti-cux1 (Santa Cruz, 1:200), rabbit anti-Tbr1 (1:500), anti-beta III Tubulin antibody (abcam, 1:1500), and mouse anti-MAP2 antibody (GeneTex, 1:100). The goat anti-rabbit or anti-mouse secondary antibodies used for immunofluorescence staining were conjugated with Alexa flour 546 (1:500), and 647 (1:500) (Thermo Fisher Scientific). Lastly, slices were counterstained with 0.5 μg/ml 4′,6-diamidino-2-phenylindole (DAPI) (Molecular Probes) for one hour, then mounted by VECTASHIELD mounting media before sealing the slides.

### Image Analysis

All images were collected with a four laser, point scanning confocal microscopy (Zeiss LSM 700), and analyzed with ZEN software (Zeiss). ImageJ software was used to quantified cells distribution in the cortex.

NeuromanticV1_6_3 (Yale.edu) software was used to trace cell bodies and apical dendrites extending from the cortical layer II/III pyramidal neurons expressing GFP (P6). Tracing images were converted by NEURON 7.5 documentation and processed by CorelDRAW X3 software.

To observe the effect of KBP knockdown in axonal projection, electroporated mouse brains at P6 and P12 were used. For analysis of callosal axon growth, a curved line was drawn along the callosal axon projection, and the green fluorescent marks within this selected line was quantified with “Line scan” in MetaMorph software. The intensity of each pixel was normalized to the average intensity of the first five pixels of the same section (Poulopoulos et al., 2019).

For analysis of neuronal morphology, cultured cortical neurons at DIV7 and DIV9 expressing GFP were randomly selected and analyzed semi-automatically with a customized MATLAB program. Circles of increasing radii (25, 50, and 75 μm) are manually centered over the cell soma by sholl analysis.

### Statistical Analysis

Statistics were presented as mean ± SEM. Student’s t and one-way ANOVA tests were used to compare differences between two and multiple experimental conditions, respectively. All statistical tests were two sided. *p* < 0.05 was considered statistically significant.

## Results

### KBP Knockdown Delayed Neuronal Migration

**FIGURE 1:**
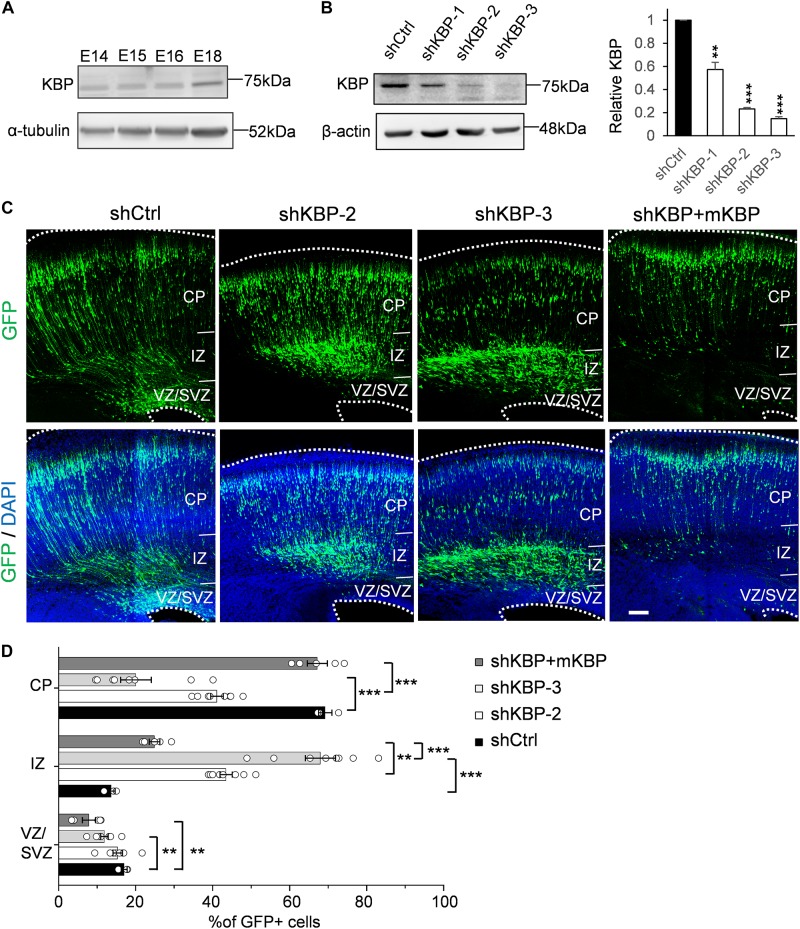
Delayed neuronal migration in the developing mouse cortex by KBP knockdown. **(A)** Western blot showing KBP expression in mouse cortical brain lysates between E14 and E18. KBP was expressed at all stages examined. **(B)** KBP expression in DIV7 cortical neurons infected with lentivirus encoding control or KBP shRNAs at DIV3. shKBP-2 and shKBP-3 showed efficient protein knockdown (>80%, *n* = 4; Error bars represent SEM. ^∗∗^*p* < 0.01, ^∗∗∗^*p* < 0.001, student’s *t*-test.) **(C)** Cell distributions in the mouse cortex 4 days after electroporation of shCtrl, shKBPs, or shKBP along with mKBP. Most of the shCtrl-electroporated GFP+ cells (green) had migrated to the CP (left panels). In contrast, most shKBP-electroporated cells were distributed in the IZ (middle panels). This cell distribution defect was rescued by expression of mKBP (right panels). Brain slices were counterstained with DAPI (blue). Bar = 100 μm. **(D)** Scatter-and-bar graph of cell distribution 4 days after IUE (shCtrl: *n* = 3; shKBP-2, shKBP-3: *n* = 4; shKBP + mKBP: *n* = 5 animals). Error bars represent SEM. ^∗∗^*p* < 0.01, ^∗∗∗^*p* < 0.001, one-way ANOVA.

To explore potential involvement of KBP in cortical development, we first examined whether KBP is expressed in the developing brain. We collected mouse cortices and extracted protein lysates at different embryonic stages between E14 and E18, during which neurogenesis and neuronal migration are highly active. Western blot showed that KBP was expressed in the developing mouse cortex at all stages examined between E14 and E18 (Figure 1A).

To identify the role of KBP in cortical development, we next investigated the effects of KBP loss-of-function by RNA interference (RNAi) in the developing brain. Three different shRNA constructs targeting KBP (shKBP-1, shKBP-2, and shKBP-3) were packaged into lentiviral particles and infected into cultured cortical neurons from E14.5 ICR mice. Among all 3 constructs, shKBP-2 and shKBP-3 (referred as shKBP hereafter) showed knockdown efficiency of >80% after 4 days (Figure 1B). We then co-electroporated neural progenitors with shKBP or shCtrl along with GFP using IUE at E14.5 and examined the distribution of electroporated cells in fixed brain slices 4 days post injection (Figure 1C). In the control brain, most shCtrl-transfected cells have migrated from VZ and reached the CP (69.2 ± 1.75%, *n* = 3 animals). In contrast, most of the shKBP-transfected cells were located in the IZ (43.5 ± 1.58%, *n* = 8 animals) and VZ/SVZ (15.3 ± 1.23%, *n* = 8 animals) and only ∼40% of the cells had reached the CP (41.1 ± 1.64%; Figures 1C,D). To avoid off-target effects of shRNA, we performed rescue experiments using shRNA-resistant mouse KBP co-electroporated with shKBP, and found the abnormal distribution phenotype was rescued (Figures 1C,D). This result demonstrated that KBP knockdown altered neural progenitor cell distribution, which can result from defects in neural progenitor cell proliferation, differentiation, and/or migration.

**FIGURE 2:**
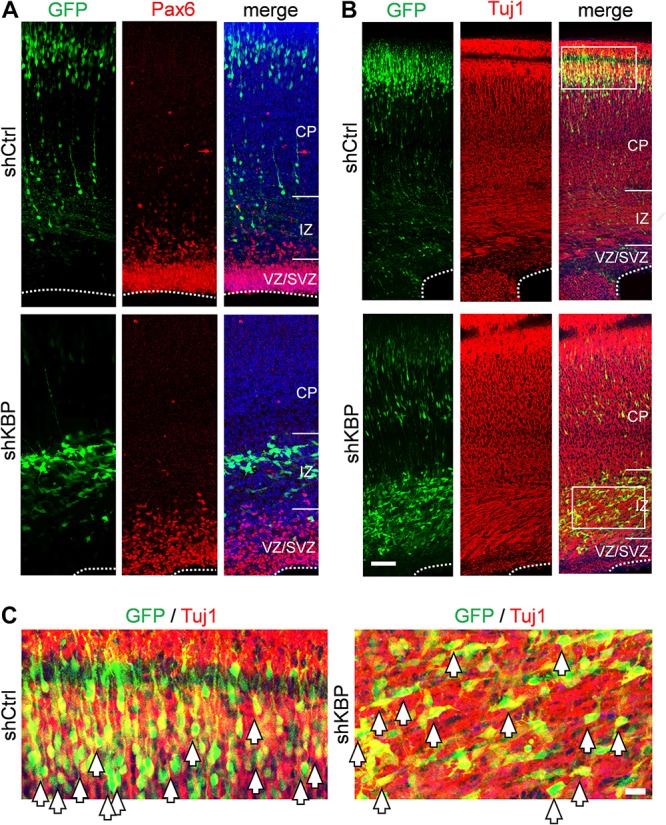
Neuronal lineage of transfected cells after IUE. **(A)** Immunostaining of Pax6 (red) in brain slices electroporated with control or KBP shRNAs. The majority of the GFP+ cells (green) were Pax-(red) both in control and KBP-KD brain. **(B)** The majority of GFP+ cells (green) were Tuj1+ (red) in both control and KBP-KD brains albeit the difference in cell distribution. Bar = 50 μm. **(C)** Zoomed images showed colocalization of GFP (green) and Tuj1 (red) signal in both shCtrl- and shKBP-electroporated neurons. Bar = 10 μm.

To determine whether the abnormal distribution can be caused by defects in differentiation, we examined the identity of KBP-knockdown cells by immunostaining of markers for neural progenitor cells Pax6 and neurons Tuj1 (Supplementary Figure S1 and Figure 2). At E16.5, we found similar percentages in GFP+ cells and Pax6+ cells (Supplementary Figure S1; shKBP: 30.76 ± 2.87% vs. shCtrl: 30.82 ± 2.69%, *n* = 3 animals). At E18.5, most shKBP-transfected cells seen in the VZ/SVZ, and IZ were Tuj1+ and Pax6− (Figure 2), indicating that these abnormally located KBP-knockdown cells have started to differentiate into neurons. This result showed that the altered distribution of KBP-knockdown cells in E18.5 cortices may not be caused by defects in neural progenitor cell differentiation.

### Cells Arrested by KBP Knockdown Differentiated Into Neurons

**FIGURE 3:**
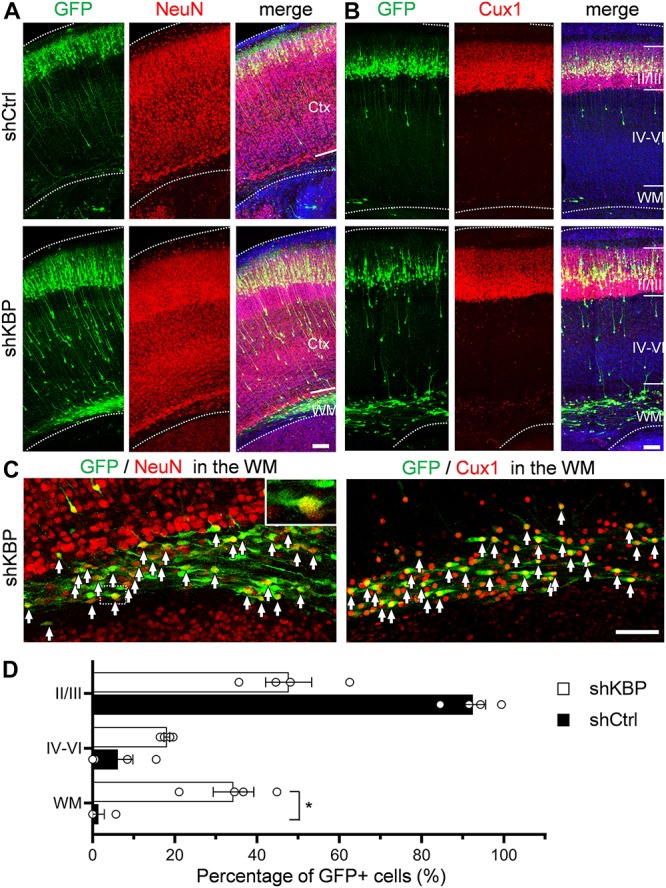
Expression of neuronal markers in KBP-KD cells in the postnatal mouse brain. **(A,B)** Immunostaining of NeuN **(A)** and Cux1 **(B)** in P6 brain slices electroporated with shCtrl or shKBP. The majority of GFP+ cells (green), including arrested cells in the WM were NeuN+ and Cux1+(red). Note that very few GFP+ cells are present in the deep cortical layers and WM at this stage. Bars = 100 μm **(C)** High magnification of the WM region in KBP-KD brain slices immunostained with NeuN and Cux1 (red). Most of the electroporated GFP+ cells (green) were also NeuN+ and Cux1+. Bar = 100 μm. **(D)** Scatter-and-bar graph showing the percentage of GFP+ cells distributed in the cortical layer II/III, layer IV–VI and WM (*n* = 4 animals in both conditions). Error bars represent SEM. ^∗^*p* < 0.05, student’s *t*-test.

To investigate whether cells arrested by KBP knockdown could catch up to the normal migration process after birth, the cell distribution of mice brains electroporated at E14.5 was examined at postnatal day 6 (P6) (Figure 3). In control brains, the majority of shCtrl-transfected cells were found in layer II/III, labeled by Cux1 (92.4 ± 3.2%; *n* = 4 animals) (Figures 3B–D). In KBP knockdown brains, about one third (34.3 ± 4.9%) of GFP+ cells remained ectopically in the WM, and only about half of GFP+ cells (47.7 ± 5.6%) had reached cortical layer II/III (Figures 3B–D). Interestingly, below layer II/III, we observed extended long leading processes in some (18.1 ± 0.7%) cells appearing as migrating neurons (Figures 3A,B), suggesting abnormalities in neuronal migration.

To characterize the cell identity of these ectopically located cells in the WM, we stained the brain slices with NeuN for differentiated post-mitotic neuronal cells and Cux1 for upper layer neurons (Figures 3A–C). Surprisingly, most of the shKBP-electroporated cells located in the WM were also NeuN+ and Cux1+ (Figure 3C). These results indicate that the cells arrested by KBP knockdown were able to differentiate into cortical neurons.

### KBP Knockdown Simplified Dendritic Arborization and Impaired Axon Extension

**FIGURE 4:**
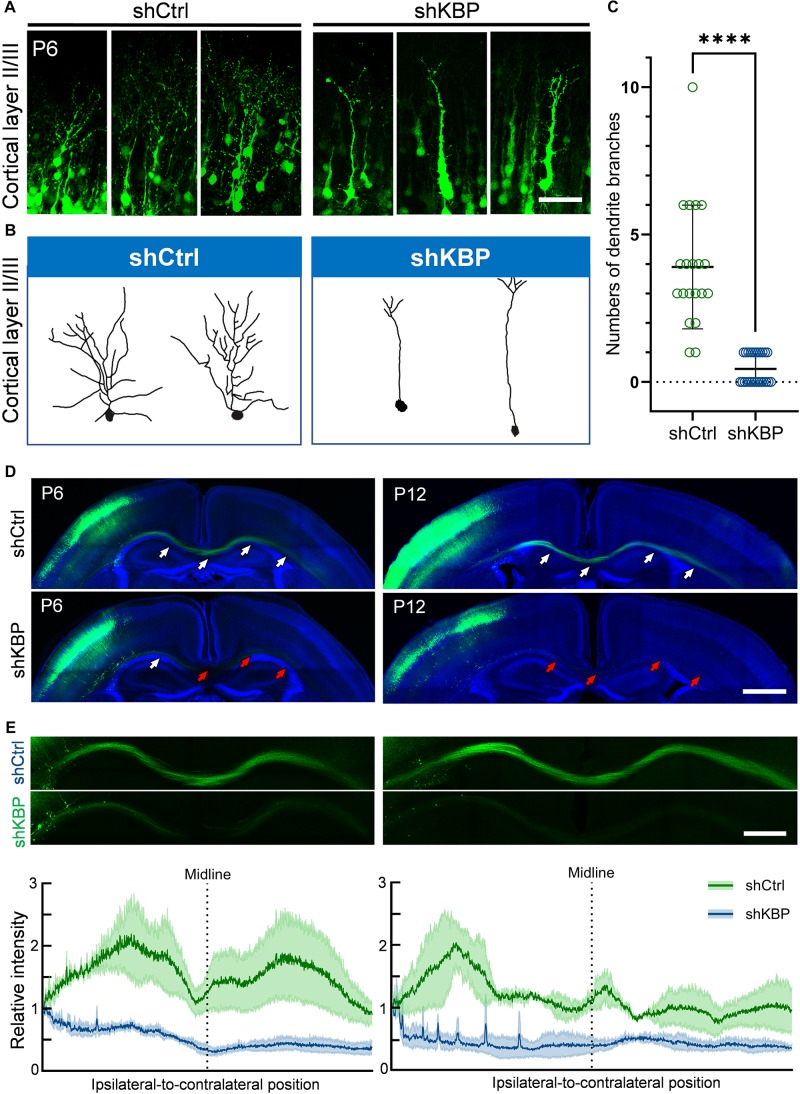
Simplified dendritic arborization and impaired axon extension in KBP-KD neurons. **(A)** Cells electroporated with control or KBP shRNAs in cortical layer II/III of P6 brain slices. Dendrites were highly branched in control GFP+ (green) neurons but the branches were simplified in KBP-KD neurons. Bar = 100 μm. **(B)** Reconstructions of layer II/III pyramidal neurons from confocal images stack showed dramatically decreased dendritic branches in KBP-KD neurons. **(C)** Scatter plot graph showing quantification of primary dendritic branches of layer II/III pyramidal neurons. Error bars represent standard deviation (SD). ^****^p < 0.0001, student’s *t*-test. **(D)** Callosal axon projection of pyramidal neurons electroporated with control or KBP shRNA in P6 (upper panels) and P12 (lower panels) mouse brain slices. GFP+ axons (green) hardly cross the midline in KBP-KD brains at P6. The effect was even more severe in P12 brains. White arrows indicate axon-projecting fibers in the transfected hemisphere, midline and contralateral cortex. Red arrows indicate the terminals of the projection axons in the KBP knockdown group. Bar = 500 μm. **(E)** Enlarged images of the callosal axon projection (top) and relative GFP intensity of the axonal projections (bottom). The intensity traces are shown as mean ± SEM from three animals. Irregular peaks on the ipsilateral side represented the somata of some GFP+ cells in the WM. Bar = 300 μm.

Interestingly, even though nearly half of KBP-knockdown neurons had migrated to upper layers of the cortex, we observed abnormalities in neurite morphology of these neurons (Figure 4). To analyze the neurite structures of these neurons, we reconstructed 3D images of GFP+ neurons from confocal image stacks by manual tracing, and showed individual processes extending from the cell soma (Figures 4A,B). Prominent apical dendrite and its branches were easily seen for comparison. We found that, while normal layer II/III pyramidal neurons exhibited well-developed dendritic trees with multiple branches, KBP-knockdown neurons contained few short branches extending from the apical dendrite (Figure 4C).

In addition to hypoplastic dendrites, we also examined the changes in callosal axon projection of pyramidal neurons layer II/III. Callosal axon projections in the mouse develop during the prenatal and early postnatal periods; therefore, we labeled axon projections with GFP at E14.5 using IUE, and collected brains at P6 and P12 (Figure 4D). In the control brain, axons projected down toward the WM, crossed the midline and extended to the contralateral side of the brain at P6. Axonal tracts extended even further into the cortex at P12. On the contrary, axons in KBP-knockdown brains only projected down to the WM and failed to extend across the midline at P6; impaired callosal axon projections were even more severe at P12, showing scarce axonal projections in the WM (Figure 4E).

**FIGURE 5:**
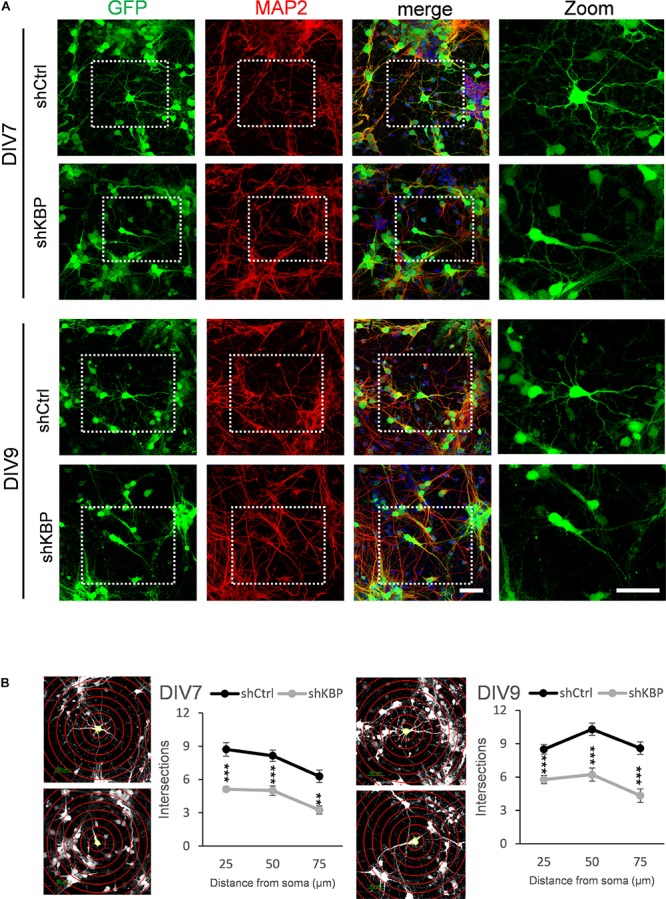
Reduced neurite complexity in cultured cortical neurons. **(A)** DIV7 and DIV9 neurons isolated from E14.5 mouse cortex and infected with lentiviruses encoding shCtrl or shKBP. Representative images of transduced cells positive to both GFP (green) and dendritic marker MAP2 (red). Right panel showed higher magnification of transfected neurons cropped in left panels. Scale bar = 50 μm. **(B)** Sholl analysis for neuronal complexity. Polyline graphs showed intersections of neurite branches at 25 μm, 50 μm, 75 μm away from the soma. KBP KD significantly reduced intersections in sholl analysis. Error bars represent SEM. ^∗∗^*p* < 0.01, ^∗∗∗^*p* < 0.001, student’s *t*-test.

To further illustrate the altered neuron morphology of KBP-knockdown mice, we cultured cortical neurons from E14.5 ICR mice (Figure 5). We then introduced lentivirus carrying shCtrl or shKBP as well as GFP into cultured neurons at DIV3. The cells were then fixed at DIV7 or DIV9 and immunostained with dendrite-specific marker MAP2 (Figure 5A). Neurite complexity was quantified using Sholl analysis on GFP+/MAP2+ neurons from confocal images (Figure 5B). We found reduced numbers of intersections from proximal to distal of dendrites in the KBP-knockdown group both at DIV7 and DIV9. These results further revealed KBP is required for dendritic branch formation.

### KBP Knockdown Increased Neuronal Apoptosis

**FIGURE 6:**
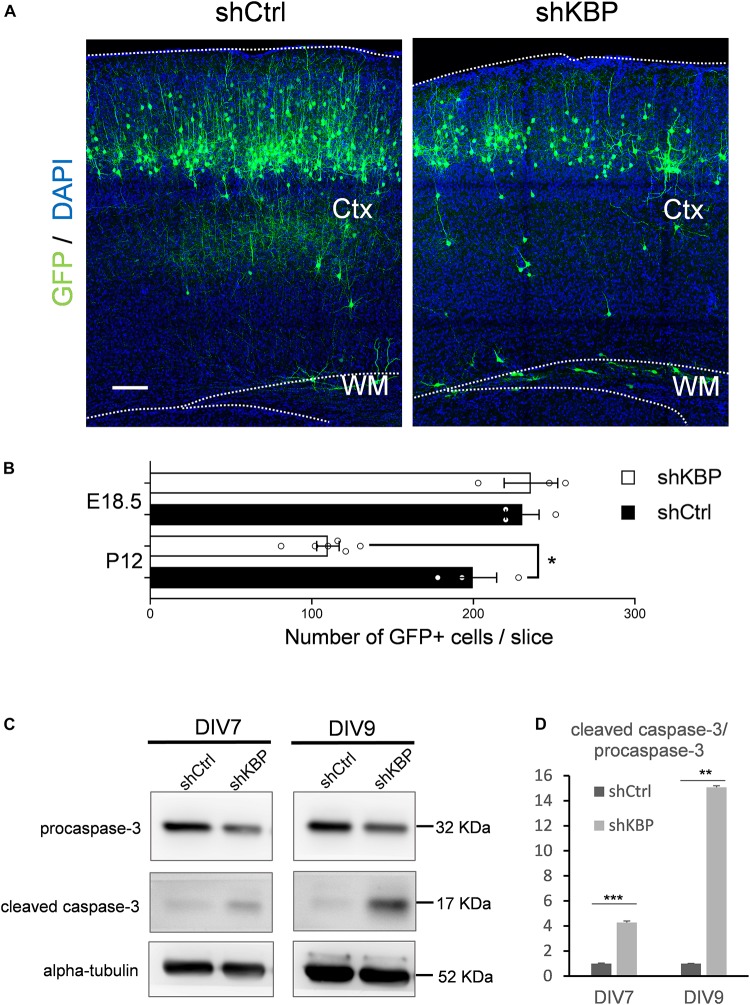
Increased neuronal apoptosis in KBP-KD brains. **(A)** P12 mouse brain slices electroporated with control or KBP KD shRNA at E14.5. Electroporated cells (green) in KBP-KD brain slices showed a decrease in the number compared to control brain slices. Scale bar = 100 μm. **(B)** Scatter-and-bar graph showing a significant decrease in GFP+ cell numbers in KBP-KD brains at P12, while cell numbers at the early stage E18.5 were similar. Error bars represent SEM. ^∗^*p* < 0.05, student’s *t*-test. **(C)** Western blot showing caspase-3 protein levels in cultured mouse cortical neurons 4 days (DIV7) and 6 days (DIV9) after infected with lentiviruses encoding control or KBP shRNA at DIV3. In KBP-KD neurons, the protein level of procaspase-3 decreased and cleaved-caspase-3 increased at both DIV7 and DIV9. **(D)** Bar graph showed normalized relative level of cleaved caspase-3 to procapase-3 at DIV7 and DIV9. KBP KD significantly increased the relative level of cleaved caspase-3 (*n* = 3). Error bars represent SEM. ^∗∗^*p* < 0.01, ^∗∗∗^*p* < 0.001, student’s *t*-test.

When collecting brain sections at P12, we also observed an apparent decrease in the number of total GFP+ cells in the KBP-knockdown brains (Figure 6A). We therefore counted the total GFP+ cells in each electroporated brain. At P12, we found the cell number was significantly decreased in the KBP-knockdown group (Figure 6B). This observation led us to speculate that loss of KBP may increase cell apoptosis since the initial number of GFP+ cells did not appear to be affected (Figures 1C,D, 6B). Therefore, we examined the apoptotic effect in cultured cortical neurons by monitoring Caspase-3 cleavage, a key process during apoptosis. E14.5 mouse cortical neurons were infected with shKBP or shCtrl lentiviruses at DIV3 and Caspase-3 was then detected at DIV7 and DIV9 by western blot analysis. We found that the relative level of cleaved caspase-3 to procapase-3 gradually increased in the KBP-knockdown group (Figures 6C,D). This result revealed that loss of KBP increases apoptosis as development progresses.

## Discussion

**FIGURE 7:**
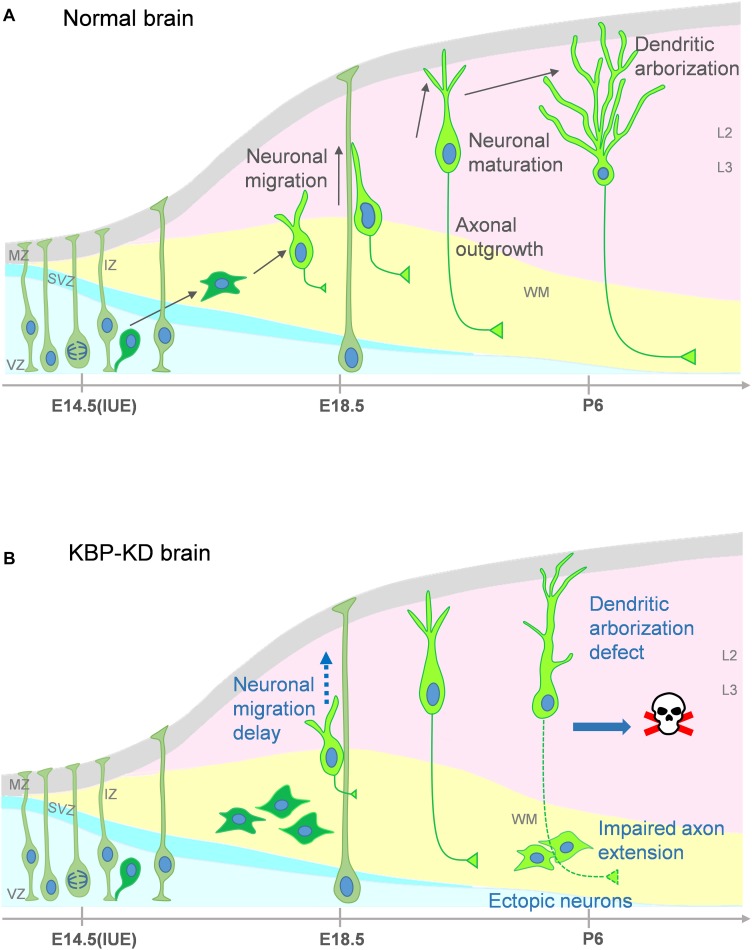
Proposed model for multiple roles of KBP in brain development and the pathogenesis of GOSHS. **(A)** In the normal brain, newborn neurons generated from neural progenitors undergo multipolar-to-bipolar transition and axongenesis at the IZ. Subsequently, they migrate radially toward the CP. As the neurons reach the CP, the leading processes become the dendrites and start to branch while the axon continues its growth to connect to other brain regions. **(B)** In the KBP-KD brain, neuronal migration is delayed and mature neurons are arrested in the white matter (WM). This phenotype correlates to PMG of GOSHS patients. Dendritic arborization of pyramidal neurons is simplified and the axon extension is impaired. These defects may contribute to intellectual disability and thinner corpus callosum formation of GOSHS patients. KBP-KD also increased neuronal apoptosis in the late postnatal period that may reduce the number of neurons, which may lead to microcephaly of GOSHS patients.

In our study, we show that loss of KBP leads to multiple dysfunction in cortical development. KBP knockdown, by IUE of shRNA, results in defects in neuronal migration (Figure 1) and leads to an ectopic layer of neurons after birth (Figure 3). Interestingly, these neurons appear to follow the normal course of differentiation and express neuronal markers Tuj1, NeuN, and Cux1 (Figures 2, 3). However, KBP-knockdown neurons exhibit simplified dendritic trees and fail to extend axonal projections through the corpus callosum to the contralateral side of the brain (Figures 4, 5). Surprisingly, KBP knockdown in developing neurons also increases apoptosis, leading to a decrease in the number of cortical neurons (Figure 6). These defects in neuronal migration, morphological maturation, and cell survival may each contribute to the clinical presentations of GOSHS in human patients (Figure 7).

### Potential Mechanisms of KBP in Neuronal Migration

The migration defect in KBP knockdown brains may be correlated to the PMG phenotype, characterized by numerous small, unfolded gyri, and disorganized lamination, in GOSHS patients (Valence et al., 2013; Squier and Jansen, 2014; Kato, 2015). Previous studies have shown that defects in neuronal migration caused MCDs, including lissencephaly and PMG (Tsai et al., 2005, 2007, 2016; Vallee and Tsai, 2006; Vallee et al., 2009; Jheng et al., 2018). For example, studies using *in utero* knockdown of genes in rat embryos have found *TUBB2B* to be essential for neuronal migration (Jaglin et al., 2009). In addition, our results showing mature neurons arrested in the WM may also recapitulate the radial columnar heterotopic neurons in the WM of PMG patients (Valence et al., 2013). Therefore, neuronal migration defects may underlie these pathological findings in the cortical malformation.

The molecular mechanisms of KBP in neuronal migration remains to be elucidated. KBP may regulate neuronal migration through its potential interaction with KIF1A and/or KIF3A, which have been shown to regulate neuronal migration (Tsai et al., 2010; Hu et al., 2013; Baffet et al., 2015; Chen et al., 2019). KIF1A was identified as a basal direction regulator of INM, and KIF3A was also found to play a role in INM regulation by the Gli2 and Cyclin D1 system. Alternatively, KBP may regulate multipolar-to-bipolar transition in the IZ before radial migration toward the CP (Cooper, 2014). Protein kinases, GTPases, cytoskeletal proteins, and adhesion molecules, such as CDK5, RhoA, kinesin 6, SCG10, and Cntn1, are also implicated in this process (Grenningloh et al., 2004; Kawauchi et al., 2006; Westerlund et al., 2011; Lin et al., 2012; Falnikar et al., 2013; Chen et al., 2018). However, the association between KBP and these proteins during this process remains elusive.

### Possible Mechanisms of KBP in Dendrite Morphogenesis

In addition, our results also revealed simplified dendritic trees and axonal extension defects in pyramidal neurons, which may contribute to intellectual disability in GOSHS patients. Dendritic arborization involving dendrite extension, addition, elongation, retraction, and pruning is a critical process that determines the synaptic input field (Arikkath, 2012). Therefore, aberrant dendrite morphogenesis such as reduced dendritic branching observed in KBP-knockdown neurons may underlie human diseases related to mental retardation.

Besides altered neuronal information input, axons for information output were also impaired in KBP-Knockdown brains. This result may reflect the thinner corpus callosum in GOSHS patients (Valence et al., 2013). The corpus callosum is the major forebrain commissure connecting right and left hemispheres, and is important for cognitive abilities. Several studies have indicated that defects in midline crossing of callosal axons by guidepost cells is one of primary cause of corpus callosum agenesis (Gelot et al., 1998; Magnani et al., 2014). Our results revealing axon extension defects in KBP-knockdown brains provide another key factor for this process.

The dendritic arborization process is regulated by multiple cellular molecules such as transcription factors, cell adhesion molecules, calcium signaling proteins and regulators of the actin cytoskeleton (Arikkath, 2012; Emoto, 2012). Previous studies indicate that KBP functions as a cytoskeleton regulator (Kevenaar et al., 2016), and thus it may play a crucial role in dendritic arborization and axonal extension. One possible mechanism of regulating microtubule dynamics is through microtubule destabilizing proteins, SCG10; this mechanism has been linked to KBP function. A study used zebrafish to demonstrate that KBP interacts with the KIF1B motor for transporting SCG10, to grow cones and complete axon extension (Drerup et al., 2016). Alternatively, KBP may also be involved in other molecular pathways to regulate axonal outgrowth, such as interaction with F-actin.

### Potential Mechanisms Responsible for Apoptosis

Finally, we also observed an increase in apoptosis in KBP-knockdown neurons, a phenotype that could reduce the number of neurons and thus brain size. Neuronal apoptosis during development is induced in several ways, such as axotomy, aberrant of cell-cycle re-entry, loss of connected neurons, oxidants, and microglial-mediated inflammation (Kristiansen and Ham, 2014; Fricker et al., 2018). Interestingly, apoptosis in KBP-knockdown neurons is accompanied by dendritic and axonal outgrowth defects, which disrupt their connections to other neurons. It is therefore possible that this loss of connection may induce neuronal apoptosis; however, this possibility remains to be determined.

## Conclusion

Our study showed that KBP dysfunction causes defects in neuronal migration, dendritic arborization, axonal extension, and neuronal apoptosis during cortical development. These cellular mechanisms may underlie the brain anomalies found in GOSHS patients.

## Data Availability Statement

All datasets generated for this study are included in the article/Supplementary Material.

## Ethics Statement

The animal study was reviewed and approved by the IACUC of National Yang-Ming University, Taipei, Taiwan.

## Author Contributions

H-YCha and J-WT conceptualized the study and wrote the manuscript. H-YCha did most of the experiments and data analysis. H-YChe, A-NT, and CL conducted the axonal analysis, Cux1 staining, Pax6 staining, and data analysis. J-WT supervised the project. All authors revised and reviewed the manuscript.

## Conflict of Interest

The authors declare that the research was conducted in the absence of any commercial or financial relationships that could be construed as a potential conflict of interest.
