# Supplementary material for: Multiple Functions of KBP in Neural Development Underlie Brain Anomalies in Goldberg-Shprintzen Syndrome
Source: Front Mol Neurosci. 2019 Nov 1;12:265. doi: 10.3389/fnmol.2019.00265 (PMC6838004; doi:10.3389/fnmol.2019.00265)
Supplement: Supplementary file 1 [file Data_Sheet_1.PDF]

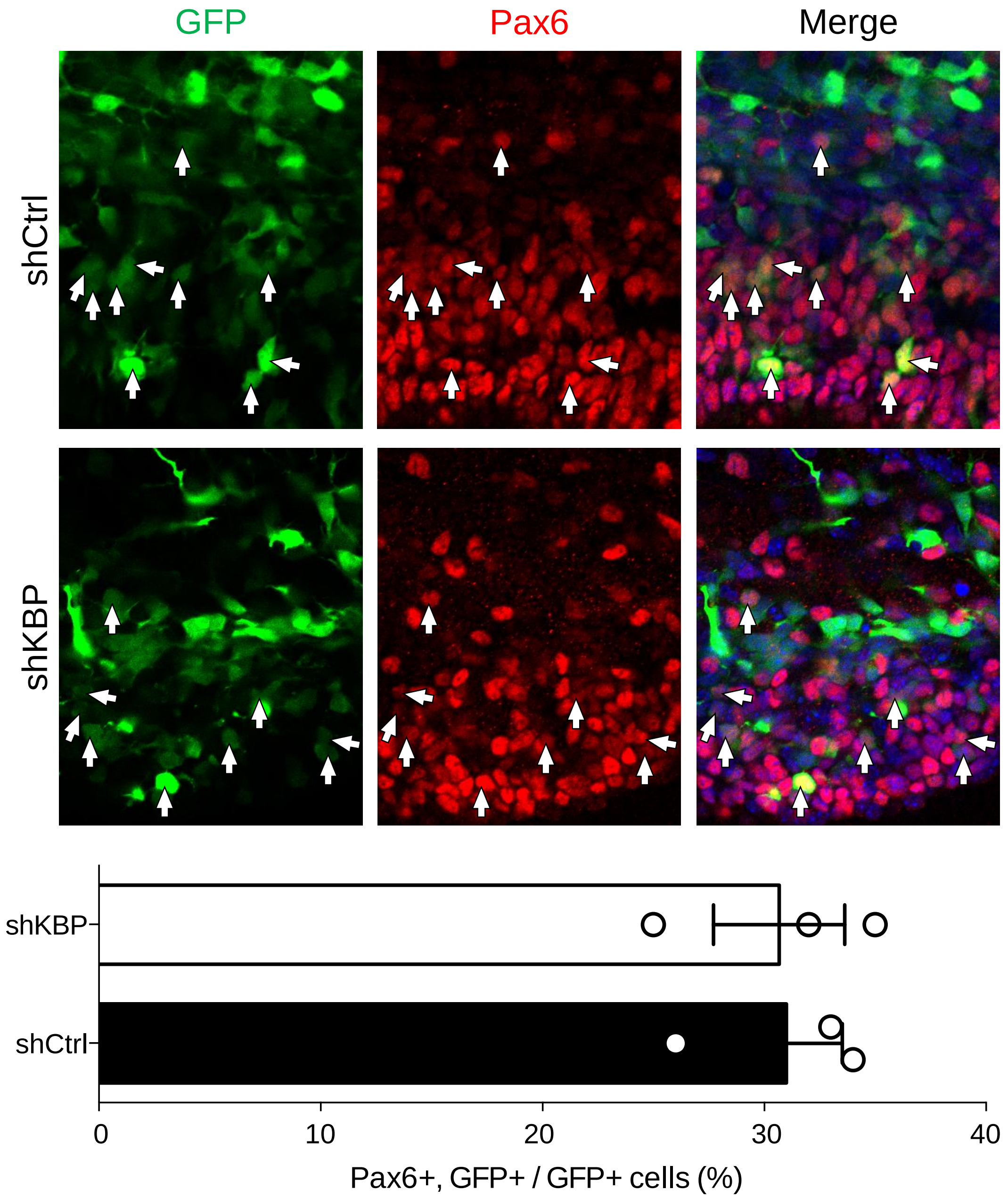

Supplementary Figure 1. Immunofluorescence staining of KBP-KD brains for the neural progenitor marker Pax6 at E16.5. Brains electroporated with shKBP or shCtrl at E14.5 were stained with Pax6 antibody at E16.5. There was no change in the percentage of Pax6+ (red) cells among electroporated GFP+ (green) cells. Error bars represent SEM. *: $p < 0.05$, student's *t*-test.
